# Assessing a digital technology-supported community child health programme in India using the Social Return on Investment framework

**DOI:** 10.1371/journal.pdig.0000363

**Published:** 2023-11-01

**Authors:** Manasi Patil, Athar Qureshi, Elina Naydenova, Anand Bang, Jay Halbert, Maarten De Vos, Poornima Nair, Madhumita Patil, Melissa M. Medvedev

**Affiliations:** 1 Feebris Ltd, Saffron Walden, United Kingdom; 2 Chetana’s Institute of Management and Research, Bandra East, Mumbai, India; 3 Society for Education, Action and Research in Community Health, Shodhgram, Chatgaon, Dhanora, Gadchiroli, Maharashtra, India; 4 Department of Pediatrics, Maidstone and Tunbridge Wells NHS Trust, Royal Tunbridge Wells, Tunbridge Wells, United Kingdom; 5 Kellogg College, University of Oxford, Park Town, Oxford, United Kingdom; 6 Stadius, Dept. of Electrical Engineering & Dept. Of Development & Regeneration, KU Leuven, Belgium; 7 Apnalaya, B/9–103 New Jaiphalwadi SRA Co-op Hsg Society, Tardeo, Mumbai, India; 8 Department of Pediatrics, University of California San Francisco, San Francisco, California, United States of America; 9 Maternal, Adolescent, Reproductive, and Child Health Centre, London School of Hygiene and Tropical Medicine, London, United Kingdom; Universität Wien: Universitat Wien, AUSTRIA

## Abstract

An estimated 5.0 million children aged under 5 years died in 2020, with 82% of these deaths occurring in sub-Saharan Africa and southern Asia. Over one-third of Mumbai’s population has limited access to healthcare, and child health outcomes are particularly grave among the urban poor. We describe the implementation of a digital technology-based child health programme in Mumbai and evaluate its holistic impact. Using an artificial intelligence (AI)-powered mobile health platform, we developed a programme for community-based management of child health. Leveraging an existing workforce, community health workers (CHW), the programme was designed to strengthen triage and referral, improve access to healthcare in the community, and reduce dependence on hospitals. A Social Return on Investment (SROI) framework is used to evaluate holistic impact. The programme increased the proportion of illness episodes treated in the community from 4% to 76%, subsequently reducing hospitalisations and out-of-pocket expenditure on private healthcare providers. For the total investment of Indian Rupee (INR) 2,632,271, the social return was INR 34,435,827, delivering an SROI ratio of 13. The annual cost of the programme per child was INR 625. Upskilling an existing workforce such as CHWs, with the help of AI-driven decision- support tools, has the potential to extend capacity for critical health services into community settings. This study provides a blueprint for evaluating the holistic impact of health technologies using evidence-based tools like SROI. These findings have applicability across income settings, offering clear rationale for the promotion of technology-supported interventions that strengthen healthcare delivery.

## Author summary

An estimated 5.0 million children under 5 years of age died in 2020, with 82% of these deaths occurring in sub-Saharan Africa and southern Asia. More than one-third of Mumbai’s population has poor access to healthcare, and child health outcomes are particularly grave among the urban poor. In this paper, we report on the implementation of a digital-technology supported programme for strengthening community-based management of childhood diseases in Mumbai’s slums. By augmenting appropriate triage and referral using an artificial intelligence (AI)-driven mobile platform operated by community health workers, the programme increased the proportion of illness episodes successfully treated in the community from 4% to 76%, subsequently reducing hospitalisations and out-of-pocket expenditures for patients and their families. Our analysis showed that the total investment for the project yielded a 13-fold social return for each Indian Rupee (INR) invested, with an annual cost per child of INR 625. This paper highlights the implementation of a novel AI-powered clinical decision-support platform to improve community-based health services. Several compelling and highly policy-relevant findings that have applicability across income settings, offering clear rationale for the promotion of interventions that strengthen primary healthcare delivery through technology-supported health programmes are discussed.

## Introduction

Globally, an estimated 5.0 million children under 5 years of age died in 2020, with 82% of these deaths occurring in sub-Saharan Africa and southern Asia [1]. Mumbai, the commercial capital of India, is known as a mega-city that attracts migrants from across the country. The high cost of living forces migrants to find shelter in slums around the city and its margins [2]. A slums is defined by the United Nations Programme on Human Settlements as, “a contiguous settlement where the inhabitants are characterised as having inadequate housing and basic services.” [3]As such, slums fulfil the bare minimum requirement of providing a roof over the head but struggle to meet necessities such as access to healthcare, adequate nutrition, clean drinking water, sanitation, and hygiene [4]. More than one-third of Mumbai’s population has limited access to medical care, and child health outcomes are particularly grave among the urban poor [5].

There are many non-profit organisations working in the Mumbai slums, but the need is vast, and resources are often insufficient to provide for all. Healthcare is fragmented, and consultations with a private doctor or hospitalisation come at a considerable cost. The high cost of prescribed medications adds another layer of financial burden. With most community members being daily- wage earners, a day of illness means loss of critical income. The M East Ward, with a population of over 800,000 (2011 census) [6], has been identified as one of the most deprived areas of Mumbai according to most health indicators, and approximately 85% of its inhabitants live in slums [7]. This ward also has the highest healthcare expenditure compared to other wards [7]. More than 50% of this expenditure is covered out-of-pocket, often via private providers [7]. With a sparsity of studies documenting healthcare-seeking behaviour, disease prevalence, and health outcomes in the community, the healthcare needs of many are currently invisible.

A multidisciplinary collaboration between a local non-governmental organisation (NGO), an academic institution, and a health technology provider was established to develop a programme for community-based management of child health in slums and evaluate its impact through an evidence-based framework for health economic assessment. This programme focused on leveraging an existing workforce, community health workers (CHWs), whose activities were augmented through an artificial intelligence (AI)-powered mobile health platform to strengthen triage and referral for children under 6 years of age, residing in M East Ward. The programme was designed to both increase early access to healthcare in the community and reduce dependence on hospitals as the provider of last resort–a healthcare delivery model which is critical for the sustainability of health systems through the COVID-19 pandemic and beyond.

Social Return on Investment (SROI) is an outcome-based framework that helps organisations measure and account for holistic value [8]. The framework was developed in the early 1990s by the Roberts Enterprise Development Fund and matured by the New Economics Foundation as a tool that helps organisations understand and quantify the social, environmental, and economic value they are creating [9]. The SROI framework helps illustrate the relationship between inputs (resources), outputs (results of the change process), outcomes (effects that occur immediately), and impacts (long-term effects of the change process per year) [10]. It has been demonstrated that the SROI framework can be used across healthcare settings, both prospectively and retrospectively [11,12]. We describe the implementation of a digital technology-based community child health platform and evaluate its holistic impact in an urban slum setting, using the SROI framework.

## Methods

### Project overview

The community health programme was designed to be implemented for a period of 12 months; however, due to COVID and lockdown restrictions, the duration was reduced to 10 months. The project was conducted as a collaboration between three organisations: Apnalaya, a non-profit organisation working in the slums of M East Ward (implementation partner); Chetana’s Institutes of Management and Research, Mumbai (academic research and evaluation partner); and Feebris Ltd., London (technology partner). A hub-and-spoke delivery model was at the core of the programme, where the anchor establishment (hub) was a community paediatrician to whom patients needing escalation of care were referred by CHWs (spokes), who were the first line of contact for the sick children.

### Geographical sites covered

A total of 12 slum clusters were included in M East Ward of the Mumbai Municipal Cooperation. Programme areas were subdivided and the CHWs followed a visitor schedule, covering a different sub-area each day of the week.

### Programme team and training

The programme team included six CHWs and one community doctor (paediatrician) recruited specifically for this project. The programme team received training in using the Mobile Health Kit to conduct digital health check-ups in the community and advise when referral was necessary. Training covered both the use of the technology and management of child health issues in the community setting. The CHWs were supervised by a project manager, providing regular feedback and refresher trainings based on the quality of data captured.

### Participant recruitment

The CHWs conducted daily door-to-door visits, with the following eligibility criteria applied to assess whether a child should receive a digital health check-up:

Inclusion criteria

- Children aged younger than 6 years
- Children experiencing common symptoms of childhood disease, including fever, cough, chest indrawing, diarrhoea, vomiting, breathing difficulties, difficulty swallowing, and signs of malnutrition
- Children flagged to a CHW because parent is alarmed by recent symptoms and/or behaviour

Exclusion criteria

- Children with trauma-related signs/symptoms (e.g., bleeding, bone fractures, lacerations, bruising); these cases were immediately escalated to hospital care by the CHWs
- Children experiencing severe symptoms (e.g., convulsions or non-arousability, inability to drink, breathlessness) who needed emergency medical care; CHWs were trained to recognise emergency signs and accompany children to the nearest tertiary-level government hospital for further management
- Children not accompanied by an adult caretaker who has the legal right to make health- related decisions about the child

### The mobile health kit

**Fig 1:**
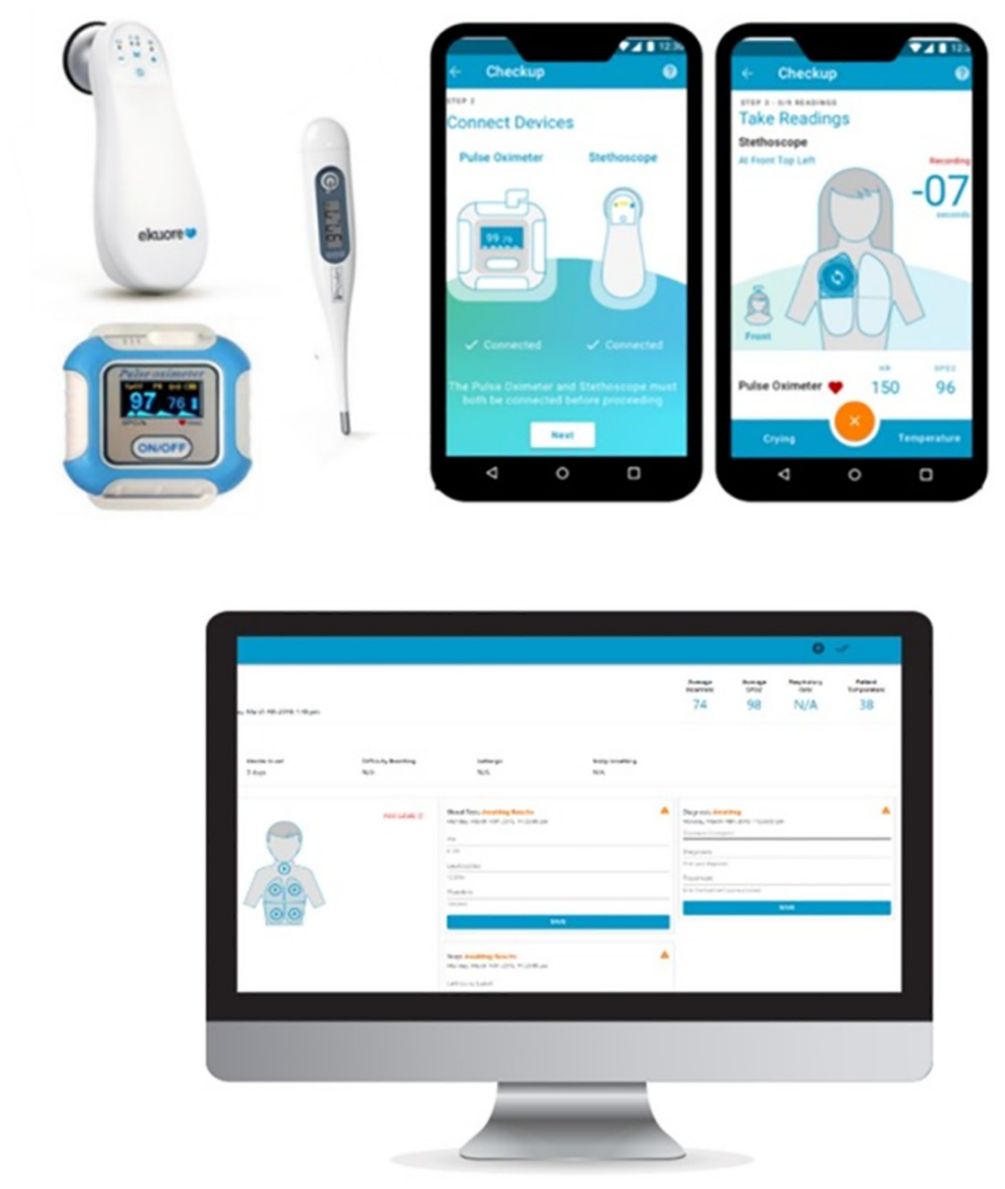
The Mobile Health Kit. (A) The Mobile Health Kit was supplied by Feebris Ltd. and included a digital stethoscope, a pulse oximeter, and an axillary thermometer, alongside an Android application preloaded on a mobile phone. The mobile application guided the CHWs through a step-by-step process of completing a questionnaire and taking measurements with the point-of-care devices. (B) All data were uploaded via the mobile application and could be viewed on a web portal by the community paediatrician. This figure has been created by EN.

The Mobile Health Kit comprised of three point-of-care (POC) devices- digital stethoscope (EKuore Pro, EKuore Medical Devices), pulse oximeter (BM2000A, Shanghai Berry Electronic Technology), and generic axillary thermometer- as well as a mobile phone (Moto G3, Motorola Mobility) with a preloaded Android application that guided the CHWs through conducting a check-up (Fig 1). The check-up could be conducted entirely offline, allowing CHWs to reach households with no/poor internet connectivity. Data were uploaded to a web portal and made available for clinical review once the CHWs reached an area with sufficient connectivity. The technology was equipped with end-to-end encryption and password-protected access for each user, ensuring full compliance with data governance and security standards (e.g., ISO 27001).

### Programme workflow

Each check-up followed a standardised structure, as outlined below. Depending on the triage result, a standardised referral process was activated to ensure children in need of clinical attention were referred to the community paediatrician at the earliest possible time.

Digital health check-up structure

- Patient registration: The CHWs collected basic data on demographic characteristics and medical history to set-up each child’s health record.
- Symptom questionnaire: The CHWs asked a series of health-related questions, such as general danger signs, respiratory complaints, diarrhoea, and vomiting, to the accompanying adult, based on the World Health Organisation (WHO) Integrated Management of Childhood Illness (IMCI) guidelines [13].
- Vital parameter measurements: The CHWs took a series of measurements with medical sensors, including temperature, heart rate, oxygen saturation, respiratory rate and auscultation of the chest using a digital stethoscope.
- Triage decision: An automatic evaluation of all symptoms against the IMCI guidelines was conducted to triage risk level (via colour-coded visualisation) and provide the CHW with a simple recommendation: a) no action; b) follow-up visit the next day; or c) referral to community doctor.

Diagnosis, referral, and treatment

**Fig 2:**
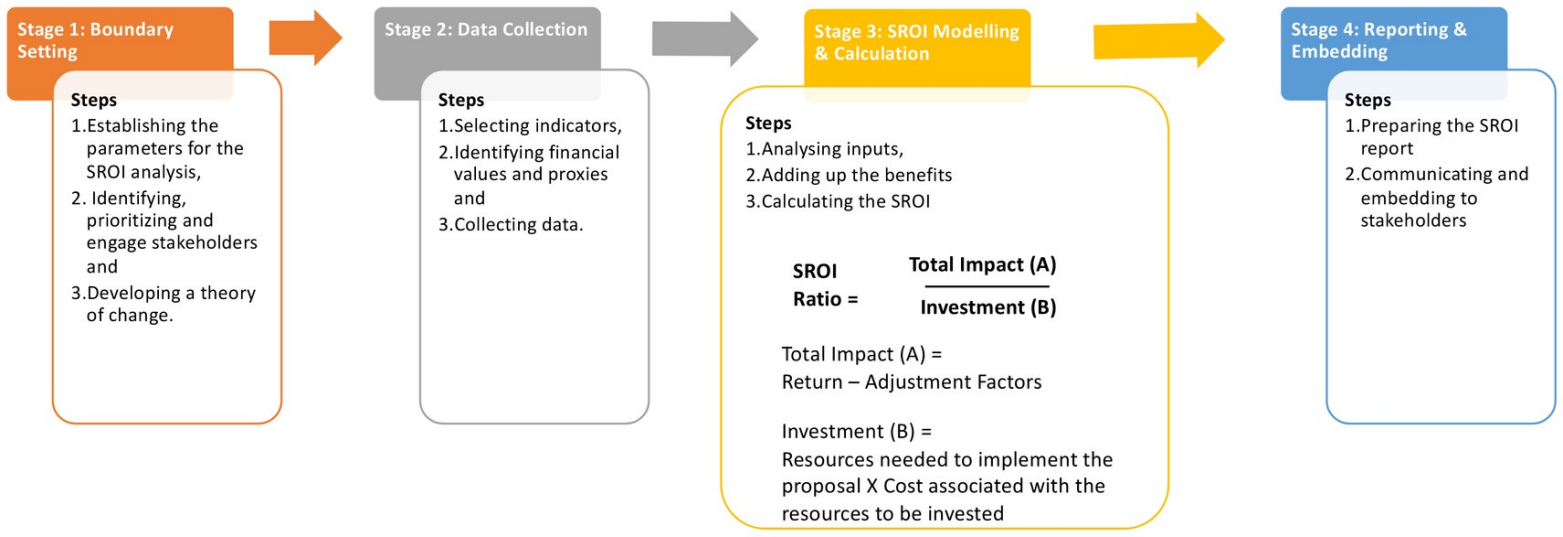
Stages of the Social Return on Investment (SROI) analysis and the process of calculating the investment, return, impact, and SROI ratio. Adapted from New Economics Foundation guide to SROI^8^.

- Clinician review: All check-ups conducted in the community could be reviewed by the community paediatrician via a digital portal that acted as an electronic medical record, capturing all health information over time. The community paediatrician was able to assess the individual vital signs as well as listen to the auscultated lung sounds of the patient via the portal. Where further tests were necessary, he could refer children to a laboratory (for blood tests/X-rays) and the CHW accompanied the parent and child to ensure continuity of care. Depending on severity, the community paediatrician either diagnosed and treated children locally or referred them further to a tertiary-level government hospital.
- Provision of treatment: Where appropriate, the community paediatrician prescribed treatment and arranged a follow-up visit.
- Hospital referral: Severe cases that could not be managed within the community were referred to a tertiary-level government hospital.
- Evaluation of impact: the impact evaluation activities were divided into four main stages:
  1. Baseline evaluation: At the beginning of the project, a survey was conducted with 3179 households to capture data on the existing health profile of the community.
  2. Programme rollout: The health programme ran for 10 months across the 12 community clusters, covering a population of approximately 5648 children.
  3. Follow-up evaluation: A survey was conducted with 284 households participating in the programme 10 months after the start of the programme to capture qualitative and quantitative data regarding its effects. A parallel survey was conducted in a control group of 627 households in a neighbouring area (that did not participate in the programme), to observe the post-intervention difference in utilisation of services between groups.
  4. SROI construction: The above three steps gave a structural and functional framework to the SROI analysis that was conducted using the New Economics Foundation guide to SROI [8], following the process illustrated in Fig 2, and the outputs of the analysis were used to calculate an SROI ratio [14].

### Research and data governance

Data were captured and stored on a Mumbai-hosted cloud (using Amazon Web Services) and fully anonymised before being shared with the research partners involved in the project. Statistical analyses were conducted using Microsoft Excel, version 16.59.

### Ethics statements

**Patient consent for publication** Not applicable.

### Ethics approval

Ethical approval was obtained from the Indian Council of Medical Research (approval number: 2018–2788). Written consent was obtained by the parent/caregiver accompanying each child participant, after they were fully informed about the purposes of the programme and research as well as any associated benefits and risks. Participants were informed that they could withdraw consent at any point.

## Results

### Key programme outcomes

A total of 5051 child health check-ups were conducted by CHWs with the Mobile Health Kit over ten months (June 1, 2019, to March 31, 2020). 3030 check-ups (59.9%) required referral to the community doctor, among which 2879 (95.0%) led to diagnosis and treatment (Table 1). Respiratory conditions accounted for 2333 (81.0%) of the diagnoses made in the community.

**Table 1:** Outcomes of the digital child health programme.

	Number (%)
Check-ups conducted, n (%)	5051 (100.0)
Check-ups performed on girls, n (%)	2475 (49.0)
Age of children (months), mean (SD)	55 (15.0)
Check-ups that required referral to the community doctor, n (%)	3030 (59.9)
Community referrals that led to diagnosis and treatment, n (%)	2879 (95.0)
Respiratory conditions, n (%)	2333 (81.0)
• Upper respiratory tract infection	2132 (91.4)
• Lower respiratory tract infection (e.g., pneumonia)	35 (1.5)
• Wheeze associated with lower respiratory tract infection	166 (7.1)
Other acute febrile illness (e.g., viral fever, dengue, malaria), n (%)	273 (9.5)
Acute gastroenteritis, n (%)	268 (9.3)
Other condition (otitis media, abscess, skin infection, acute malnutrition), n (%)	5 (0.2)

SD = standard deviation.

**Fig 3:**
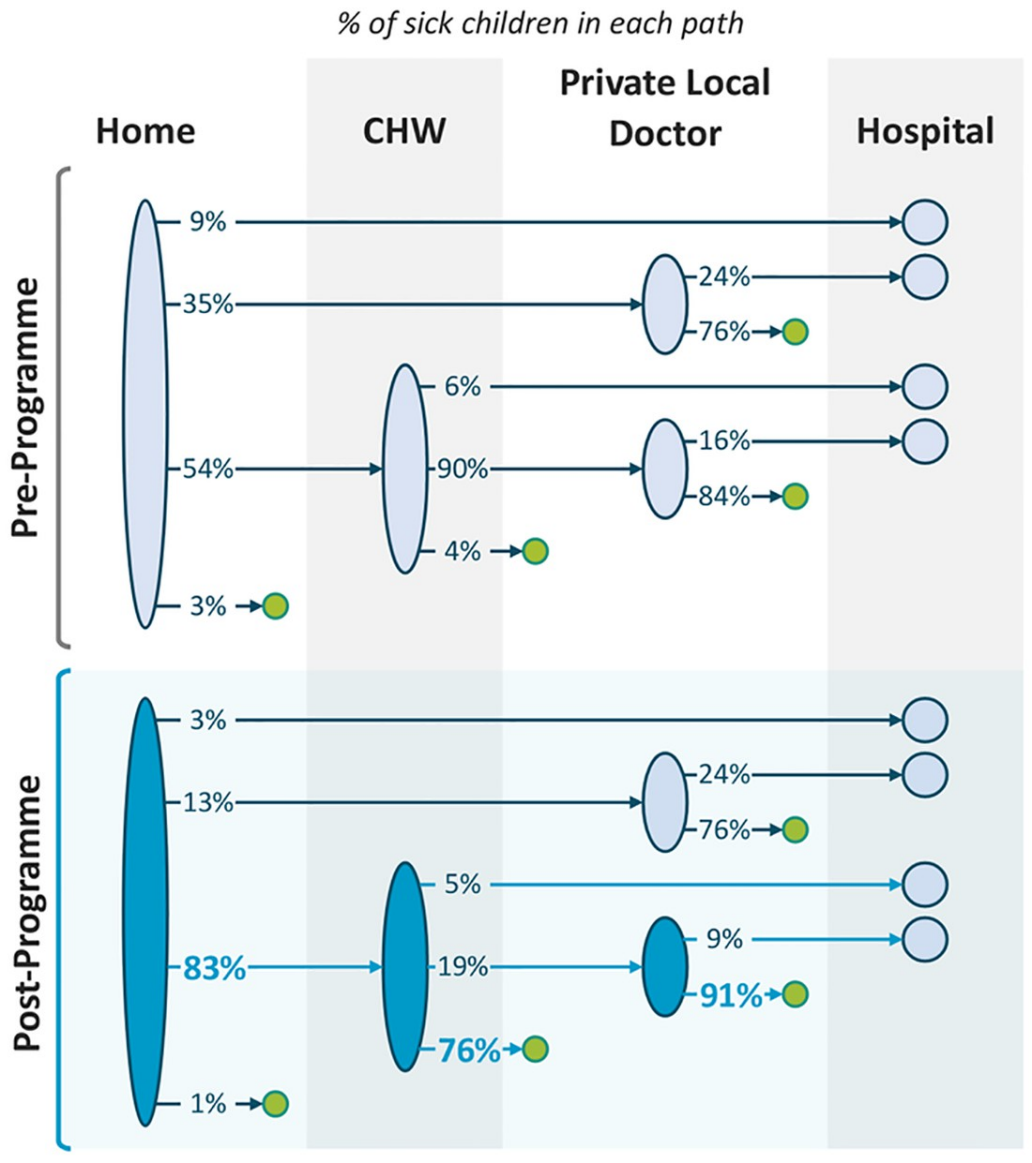
Changes in treatment pathways following implementation of the digital child health programme. The programme increased the proportion of sick children able to be seen at home by community health workers (CHW) from 54% to 83%, and the proportion of illness episodes successfully treated in the community (without the need for out-of-pocket expenditure on private consultations or hospital visits) from 4% to 76%.

The pre- and post-intervention surveys showed that consultations with a private doctor reduced from 82.8% to 19.0% and those with a hospital reduced from 34.8% to 7.0%. There was a decrease in parents reporting a loss in daily wages from 9.7% to 4.2%. (Tables 2 and 3). The programme increased the proportion of sick children seen at home by CHWs from 54% to 83%, and the proportion of illness episodes successfully treated in the community from 4% to 76% (Fig 3).

**Table 2:** Outcomes of pre-intervention baseline survey.

	Pre-intervention baseline survey*
Consultation with a CHW, n (%)	460 (54.6)^†^
Consultation with a private doctor, n (%)	698 (82.8)^†^
Consults with private doctor per episode of illness, median (IQR)	2 (1–2)
Cost of consultation with private doctor (INR), median (IQR)	75 (50–123)
Cost of transport to private doctor (INR), median (IQR)	0 (0)
Cost of medication prescribed by private doctor (INR), median (IQR)	160 (90–300)
Consulted a hospital, n (%)	240 (34.8)^‡^
Consults with hospital per episode of illness, median (IQR)	2 (1–3)
Cost of hospital consultation (INR), median (IQR)	40 (10–118)
Cost of transport to hospital (INR), median (IQR)	100 (0–150)
Cost of medication prescribed by hospital (INR), median (IQR)	200 (100–375)
Led to hospitalisation, n (%)	27 (3.9)^‡^
Duration of hospitalisation (days), median (IQR)	4 (3–6)
Parents reported experiencing loss in daily wage, n (%)	67 (9.7)^‡^

CHW = community health worker. INR = Indian Rupee. IQR = interquartile range.

*Participants were asked to report their experience of seeking healthcare in the one month prior to surveying.

^†^The denominator for this data is 843.

^‡^The denominator for this data is 689.

**Table 3:** Outcomes of post-intervention follow-up survey and parallel survey in the control group.

	Post-intervention follow-up–survey (n = 284)	Parallel survey in control group (n = 627)
Consultation with a CHW (non-project CHW), n (%)	62 (21.8)	94 (15.0)
Consultation with a private doctor, n (%)	54 (19.0)	356 (56.8)
Consulted a hospital, n (%)	20 (7.0)	210 (33.5)
Led to hospitalisation, n (%)	6 (2.1)	69 (11.0)
Duration of hospitalisation (days), mean (SD)	6 (3.0)	9 (18.0)
Daily wage loss, n (%)	12 (4.2)	190 (30.3)
Parents reported feeling less concerned about child’s health as a result of programme, n (%)	281 (98.9)	N/A

CHW = community health worker. SD = standard deviation. N/A = not applicable.

### Quantifying impact via SROI analysis

**Fig 4:**
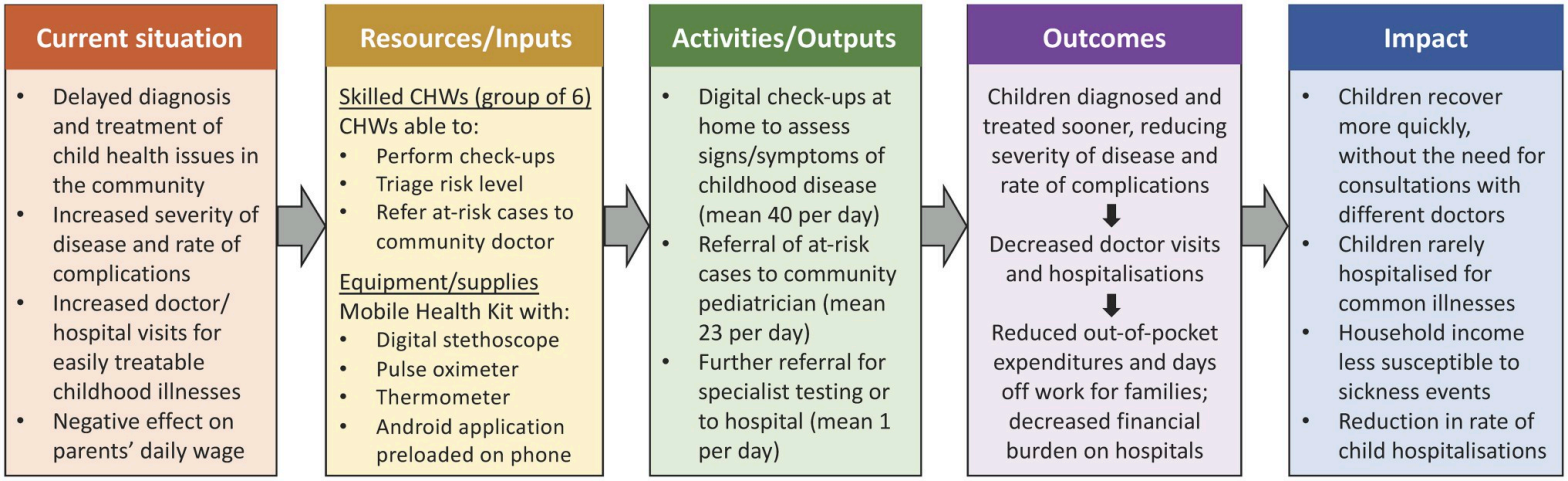
Theory of change for the digital child health programme. CHW = community health worker.

To evaluate the overall impact of the programme, the benefits to different stakeholders were examined. Described below are the stages of the SROI analysis:

Stage 1—Boundary setting and impact mapping: The baseline study helped establish the scope, identify the stakeholders, and develop the theory of change (Fig 4) [15]. Stakeholders identified and prioritised for the intervention were as follows: a) direct beneficiaries: child and family; b) community workforce: CHWs and doctor; c) wider health system: health facilities/hospitals and healthcare providers.
Stage 2—Data collection: The stage of data collection comprised of three components: a) Identification of indicators for each of the outcomes; b) assigning of financial values or, where necessary, proxies to these indicators; and c) tabulation and analysis of relevant data from the 10- month programme, alongside the baseline and follow-up surveys.
Stage 3—Modelling and calculations: The programme accounted for five main inputs (Table 4).

**Table 4:** Inputs reported for the full 10 months of the programme.

	Value (INR)
Six CHWs	900,000
Community doctor	428,571
Healthcare costs (lab tests, transport for referrals, medical support for poor patients)	453,000
Programme administration costs (programme manager, local research organiser, administrator)	683,700
Technology costs (including CHW training and equipment maintenance) *	167,000
***Total inputs and investments***	***2*,*632*,*271***

CHW = community health worker. INR-Indian Rupee.

*The technology costs only included the cost of the hardware; no software costs were charged by the software developer, Feebris.

In calculating the benefits, quantity was taken as the amount of change per child per month and value of financial proxy as the value of change per child per month.

Key assumptions while calculating the total project value (Table 5) included the following:

1. Medication costs remain the same.
2. Out-of-pocket expenditure for families reduces to INR 0 since both CHW check-up and community doctor consultation were provided for free.
3. Cost of travel reduces to INR 0 since CHW conducts check-ups at home.
4. Attribution (the extent to which outcomes were influenced by others), deadweight (what would have happened without the programme), and drop-off (outcome reduction in future years) were all assumed to be zero. This was deemed appropriate as the control group showed rates worse or equivalent to the baseline.

**Table 5:** Calculating the total impact.

Outcomes	Indicator*	Quantity*^	Project duration (months)	Number of children	Value of financial proxy (INR)	Total value or total impact (INR)
		(A)	(B)	(C)	(D)	(A) X (B) X (C) X (D)
1. Children receive diagnosis and treatment sooner, which reduces severity of disease and rate of complications	Reduced spend on medication (private doctor)	0.66 (reduction from 85% to 19%)	10	5051	320 (median cost of medication prescribed by a private local doctor for 2 consults)	10,667,712
	Reduced spend on medication (hospital)	0.21 (reduction from 29% to 8%)	10	5051	400 (median cost of medication prescribed by a hospital visit for 2 consults)	4,242,840
	Number of consultations with private doctor	0.66 (reduction from 85% to 19%)	10	5051	150 (median cost of 2 consults with a private local doctor)	5,000,490
	Number of consultations with hospital	0.21 (reduction from 29% to 8%)	10	5051	80 (median cost of 2 hospital consultations)	848,568
	Number of trips to private doctor	0.66 (reduction from 85% to 19%)	10	5051	0 (median cost of transport to a private doctor)	0
	Number of trips to hospital	0.21 (reduction from 29% to 8%)	10	5051	200 (median cost of transport for 2 visits to the hospital)	2,121,420
2. Reduction in disease severity means treatment can be delivered in the community, decreasing family (out-of-pocket) expenditure	Number of hospital stays annually per 1000 children	0.02 (reduction from 4% to 2%)	10	5051	5,438 (mean cost of hospital stay covered by out-of-pocket payments)^19^	5,493,872
3. Reduction in visits to doctor and hospital means fewer days away from work for parents	Daily wages lost due to child illness per month	0.06 (reduction from 10% to 4%)	10	5051	791 (wage loss due to hospitalisation for 4 days)	2,398,344
4. Reduction in severity means treatment can be delivered in the community and hospitalisation rates fall, reducing financial burden on government hospitals	Number of hospital stays annually per 1000 children	0.02 (reduction from 4% to 2%)	10	5051	3,626 (mean cost of hospitalisation covered by the health system)^19^	3,662,581
***Total present value or total impact***						***34*,*435*,*827***

INR = Indian Rupee.

*Reduction in the percentage of children who get ill every month compared to baseline.

^Amount of change per child per month.

The total inputs or investments for the project was INR 2,632,271, leading to an SROI ratio of 13. The annual cost of the programme per child was INR 625.

Stage 4—Reporting and embedding: This paper has been written to make the findings of this analysis and the impact of the programme across the different stages of the patient journey accessible for a wider audience.

## Discussion

Evidence on the use and impact of digital technology-supported health interventions in low- and middle-income country (LMIC) settings is sparse, especially in urban slums like M East Ward. This paper offers an evidence-based approach to evaluate the impact of a digital health intervention designed to improve access to healthcare for children, factoring in the fragmented nature of healthcare provision in this setting. The key objectives of the programme were to increase early access to healthcare in the community and reduce the dependence on hospitals as provider of last resort.

As seen from the baseline survey, accessing healthcare can be challenging for people residing in the community, often involving multiple visits with CHWs, private doctors, and trips to the hospital. Each episode of illness is therefore often related to significant out-of-pocket expenditure for the family, costing a major part of the household income. By embedding a digital health platform into the workflow of CHWs and a community paediatrician, this project provided insights into the potential of augmenting an existing workforce to improve access to healthcare. The changes in the patient journey, evidenced through a baseline and follow-up comparison demonstrated that, a significant number of illness episodes could be resolved within the community and consultations with private doctors could be reduced, resulting in monetary savings. The community-based intervention was also recorded to reduce hospitalisations; this is likely to be due to early diagnosis and intervention, reducing exacerbations. In addition to these health-economic benefits, the intervention was seen to reassure parents who reported trust in the programme and reduced concern for their child’s health during an illness episode.

There are several methods typically used to calculate social value, including cost-effective analysis (CEA), cost-utility analysis (a sub-type of CEA), and cost-benefit analysis, but these have been found to suffer from imprecision/inaccuracy when it comes to complex settings and interventions [16]. In this study, the SROI framework proved to be suitable for identifying and quantifying health economic outcomes for the key stakeholders. Moreover, the essence of the SROI method is its emphasis on developing a theory of change to understand the direction of the inputs and the outcomes of any project. The SROI analysis showed that the total investment for the project yielded a 13-fold (ratio 13:1) social return for each INR invested, with an annual cost per child of INR 625. A systematic review of studies using SROI methodology to assess the value of public health interventions found ratios ranging from 1.5:1 to 65:1 [10]. We aimed for a conservative approach, neither overestimating the potential social return nor underestimating the required expenses to achieve it. When computing total benefits, we excluded benefits to human resources, as these were difficult to quantify over the duration of the programme. However, the CHWs provided feedback on a continuous basis which was used to inform changes to the technology and service delivery and improve parents’ experience of the same.

The reduced time to diagnosis, high proportion of children successfully treated in the community, and reduced dependence on hospital care reported in this study help strengthen the case for community-based interventions, especially in a country like India, which has approximately 1,000,000 CHWs [17]. Studies have shown that CHWs have the potential to boost healthcare access and support delivery of primary health services in LMIC settings [18, 19]. A trial of a CHW-led digital health intervention for cardiovascular disease in India reported local variation in effectiveness, mediated by community trust, acceptability of CHWs’ new roles, and their connection to community members and qualified healthcare providers [20]. Equipped with clinical decision-support tools like the one used in this study, CHWs can be upskilled to triage risk level and refer at-risk cases within the community, improving efficiency and strengthening the health system.

This study had several limitations. Some community members did not benefit from the services offered by the programme for reasons including migration, cultural biases, and preference for traditional health practices. Due to time and funding limitations, we were unable to assess the impact of seasonality or disease trends related to the disruption in service delivery during the COVID-19 lockdown. A control group was used to inform any deadweight and attribution effects for the SROI analysis, rather than acting as a direct comparator. The continuation of services and the employment of healthcare staff following project completion would depend on the availability of NGO funds or the adoption of this intervention by the government health system. Finally, the technology costs did not include a software licence, as the software was provided free of charge. Nevertheless, this evaluation can be used by funders to decide what software costs are reasonable to achieve a high ROI.

## Conclusion

In this study investigating the benefits of an AI-guided digital health intervention for vulnerable children living in urban slums, we found that the programme dramatically improved the CHWs’ ability to detect health issues in the community and facilitate appropriate referral. This study provides an initial blueprint for evaluating the holistic impact of digital health technologies using evidence-based tools like SROI. The findings have applicability across income settings, offering clear rationale in favour of strengthening community-first detection and management of disease to improve health outcomes and alleviate pressure on hospital infrastructure. To realise the goal of universal health coverage, it is essential that healthcare and technology communicate seamlessly.

## Supporting information

S1 File SROI Calculation INR. (PDF)

S2 File SROI Calculation USD. (PDF)
