# Supplementary material for: Assessing a digital technology-supported community child health programme in India using the Social Return on Investment framework
Source: PLOS Digit Health. 2023 Nov 1;2(11):e0000363. doi: 10.1371/journal.pdig.0000363 (PMC10619782; doi:10.1371/journal.pdig.0000363)
Supplement: S1 File — (PDF) [file pdig.0000363.s001.pdf]

| Stage 1 | | Stage 2 | | | Stage 3 | | | | | | | | | | | Stage 4 | | | |
|---|---|---|---|---|---|---|---|---|---|---|---|---|---|---|---|---|---|---|---|
| **Stakeholders**<br>*Who do we have an effect on? Who has an effect on us?* | **Intended/ unintended changes**<br>*What changes for them?* | **Inputs**<br>Description<br>*What do they invest?* | Value (INR) | **Outputs**<br>*Summary of activity in numbers* | **Outcomes**<br>Description<br>*How do we describe the change?* | Indicator<br>*How do we measure it?* | Source<br>*Where did we get the information from?* | Quantity<br>*How much change was there?*<br>***Per child per month*** | Duration<br>*How long does it last?* | Financial proxy<br>*What proxy do we use to value change?* | Value INR<br>*What is the value of change?*<br>***Per child per month*** | Source<br>*Where did we get this information from?* | Assumptions<br>*What assumptions went into the calculation of total value?* | Going Forward<br>*How can we make these calculations more accurate and thorough?* | Total Value INR<br>*Quantity x program duration x # of children* | **Deadweight**<br>%<br>*What would have happened without the activity?* | **Attribution**<br>%<br>Who else contibuted to the change? | **Drop Off**<br>%<br>Does the outcome drop off in future years? | **Impact INR**<br>Total Value |
| **Children** | Children don't experience severe conditions as much | time and money | **0** | Daily health checkups with children in the community (avg. 40 checkups per day) | Through the community service, children receive diagnosis & treatment sooner which reduces complications and severity of disease leading to fewer appointments and travel | Reduced spend on medication (private doc) | Baseline research | **0.66** | | Monthly spend on medication | **320** | Baseline research | Assume medication costs stay the same | How to cover NGO covering medication costs? | **1,06,67,712** | 0% | 0% | 0% | **1,06,67,712** |
| | | | | | | Reduced spend on medication (hospital) | | **0.21** | | | **400** | | | | **42,42,840** | 0% | 0% | 0% | **42,42,840** |
| | | | | | | # consultations with private doctor | Baseline research | **0.66** | | Average cost of consultations | **150** | Baseline research | Assume cost of consultation reduces to £0 | | **50,00,490** | 0% | 0% | 0% | **50,00,490** |
| | | | | | | # consultations with hospital | | **0.21** | | | **80** | | | | **8,48,568** | 0% | 0% | 0% | **8,48,568** |
| | | | | | | # trips to private doctor | | **0.66** | | | **0** | | | | **0** | 0% | 0% | 0% | **0** |
| | | | | | | # trips to hospital | Baseline research | **0.21** | | Monthly cost of travel | **200** | Baseline research | Assume cost of travel reduces to £0 | | **21,21,420** | 0% | 0% | 0% | **21,21,420** |
| | Children do not get hospitalised as much | time and money | **0** | Referrals for further tests | Reduction in severity means that treatment can be delivered in the community and hospitalisation rates fall | Number of hospitalisations annually per 1000 children | Baseline research | **0.02** | | Cost of average hospitalisation | **5,438** | External Research (Elina slide deck) | | | **54,93,872** | 0% | 0% | 0% | **54,93,872** |
| **Children's families** | Families lose fewer daily wages due to seeking care for child | time | 0 | | Reduction in visits to doctor and hospital means fewer days away from work | Daily wages lost due to child illness per month | Baseline Research | **0.06** | | Daily wages lost due to child illness per month | **791** | Baseline research | Assumes that the percentage of patients that present to a doctor and to a hospital >2 times a month, both drop to 50. and the appointments means they miss 1 working day of 1 persons wages per month | | **23,98,344** | 0% | 0% | 0% | **23,98,344** |
| **Healthcare worker** | Higher staff retention | time | **9,00,000** | | Being able to make a bigger difference, whilst being less stretched and paid more leads to higher staff retention | Lower staff turnover rates | NA | NA | | Cost of replacing employees that leave | **0** | NA | NA | | **0** | 0% | 0% | 0% | **0** |
| | Less time per assessment | time | | | The technology removes barriers for assessments meanings they can be done quicker, freeing up worker hours | Less time (on average) per assessment | NA | NA | | Hourly rate of healthcare worker | **0** | NA | NA | | **0** | 0% | 0% | 0% | **0** |
| **Community doctor** | Will see less cases that don't need clinical intervention | time | **4,28,571** | | Doctor focuses on cases which need clinical intervention | % of cases at community clinic who do not need doctor intervention FP/(TP+FP) | NA | NA | | Hourly rate of clinician | **0** | NA | NA | | **0** | 0% | 0% | 0% | **0** |
| **Healthcare provider (e.g. hospital)** | Cost Only | time | **11,36,700** | | NA | NA | NA | NA | | NA | **0** | NA | NA | | **0** | 0% | 0% | 0% | **0** |
| **Health system** | Reduced hospitalizations | time and money | **0** | | Reduction in severity means that treatment can be delivered in the community and hospitalisation rates fall | Number of hospitalisations annually per 1000 children | Baseline research | **0.02** | | Cost of average hospitalisation | **3,626** | External Research (Elina slide deck) | | | **36,62,581** | 0% | 0% | 0% | **36,62,581** |
| **Payer** | Cost Only (Technology) | money | **1,67,000** | | NA | NA | NA | NA | | NA | **0** | | NA | | **0** | 0% | 0% | 0% | **0** |
| | | **TOTAL** | **26,32,271** | | | | | | | | | | | | | | | | |

| | |
|---|---|
| **TOTAL PRESENT VALUE** | **3,44,35,827** |
| **NET PRESENT VALUE** | **3,18,03,555** |
| **SOCIAL RETURN £ PER £** | **13** |
