# Supplementary material for: Assessing a digital technology-supported community child health programme in India using the Social Return on Investment framework
Source: PLOS Digit Health. 2023 Nov 1;2(11):e0000363. doi: 10.1371/journal.pdig.0000363 (PMC10619782; doi:10.1371/journal.pdig.0000363)
Supplement: S2 File — (PDF) [file pdig.0000363.s002.pdf]

Rupees to Dollars Conversion	0.012

Stage 1		Stage 2			Stage 3											Stage 4			
**Stakeholders** *Who do we have an effect on? Who has an effect on us?*	**Intended/ unintended changes** *What changes for them?*	**Inputs** Description *What do they invest?*	Value (INR)	**Outputs** *Summary of activity in numbers*	**Outcomes** Description *How do we describe the change?*	Indicator *How do we measure it?*	Source *Where did we get the information from?*	Quantity *How much change was there?* ***Per child per month***	Duration *How long does it last?*	Financial proxy *What proxy do we use to value change?*	Value INR *What is the value of change?* ***Per child per month***	Source *Where did we get this information from?*	Assumptions *What assumptions went into the calculation of total value?*	Going Forward *How can we make these calculations more accurate and thorough?*	Total Value INR *Quantity x program duration x # of children*	**Deadweight** % *What would have happened without the activity?*	**Attribution** % Who else contibuted to the change?	**Drop Off** % Does the outcome drop off in future years?	**Impact USD** Total Value
**Children**	Children don't experience severe conditions as much	time and money	**0**	Daily health checkups with children in the community (avg. 40 checkups per day)	Through the community service, children receive diagnosis & treatment sooner which reduces complications and severity of disease leading to fewer appointments and travel	Reduced spend on medication (private doc)	Baseline research	**0.66**		Monthly spend on medication	**4**	Baseline research	Assume medication costs stay the same	How to cover NGO covering medication costs?	**1,28,013**	0%	0%	0%	**1,28,013**
						Reduced spend on medication (hospital)		**0.21**			**5**				**50,914**	0%	0%	0%	**50,914**
						# consultations with private doctor	Baseline research	**0.66**		Average cost of consultations	**2**	Baseline research	Assume cost of consultation reduces to £0		**60,006**	0%	0%	0%	**60,006**
						# consultations with hospital		**0.21**			**1**				**10,183**	0%	0%	0%	**10,183**
						# trips to private doctor		**0.66**			**0**				**0**	0%	0%	0%	**0**
						# trips to hospital	Baseline research	**0.21**		Monthly cost of travel	**2**	Baseline research	Assume cost of travel reduces to £0		**25,457**	0%	0%	0%	**25,457**
	Children do not get hospitalised as much	time and money	**0**	Referrals for further tests	Reduction in severity means that treatment can be delivered in the community and hospitalisation rates fall	Number of hospitalisations annually per 1000 children	Baseline research	**0.02**		Cost of average hospitalisation	**65**	External Research (Elina slide deck)			**65,926**	0%	0%	0%	**65,926**
**Children's families**	Families lose fewer daily wages due to seeking care for child	time	0		Reduction in visits to doctor and hospital means fewer days away from work	Daily wages lost due to child illness per month	Baseline Research	**0.06**		Daily wages lost due to child illness per month	**9**	Baseline research	Assumes that the percentage of patients that present to a doctor and to a hospital >2 times a month, both drop to 50. and the appointments means they miss 1 working day of 1 persons wages per month		**28,780**	0%	0%	0%	**28,780**
**Healthcare worker**	Higher staff retention	time	**10,800**		Being able to make a bigger difference, whilst being less stretched and paid more leads to higher staff retention	Lower staff turnover rates	NA	NA		Cost of replacing employees that leave	**0**	NA	NA		**0**	0%	0%	0%	**0**
	Less time per assessment	time	**0**		The technology removes barriers for assessments meanings they can be done quicker, freeing up worker hours	Less time (on average) per assessment	NA	NA		Hourly rate of healthcare worker	**0**	NA	NA		**0**	0%	0%	0%	**0**
**Community doctor**	Will see less cases that don't need clinical intervention	time	**5,143**		Doctor focuses on cases which need clinical intervention	% of cases at community clinic who do not need doctor intervention FP/(TP+FP)	NA	NA		Hourly rate of clinician	**0**	NA	NA		**0**	0%	0%	0%	**0**
**Healthcare provider (e.g. hospital)**	Cost Only	time	**13,640**		NA	NA	NA	NA		NA	**0**	NA	NA		**0**	0%	0%	0%	**0**
**Health system**	Reduced hospitalizations	time and money	**0**		Reduction in severity means that treatment can be delivered in the community and hospitalisation rates fall	Number of hospitalisations annually per 1000 children	Baseline research	**0.02**		Cost of average hospitalisation	**44**	External Research (Elina slide deck)			**43,951**	0%	0%	0%	**43,951**
**Payer**	Cost Only (Technology)	money	**2,004**		NA	NA	NA	NA		NA	**0**		NA		**0**	0%	0%	0%	**0**
		**TOTAL**	**31,587**																

**TOTAL PRESENT VALUE**	**4,13,230**
**NET PRESENT VALUE**	**3,81,643**
**SOCIAL RETURN £ PER £**	**13**
